# Human T-lymphotropic virus type 1 p30 interacts with REGγ and ATM (Ataxia Telangiectasia Mutated) to promote cell survival

**DOI:** 10.1186/1742-4690-8-S1-A202

**Published:** 2011-06-06

**Authors:** Rajaneesh Anupam, Antara Datta, Nadine Bowden, Nikolozi Shkriabai, Mamuka Kvaratskhelia, Michael Lairmore

**Affiliations:** 1 Department of Veterinary Biosciences, The Ohio State University, Columbus, Ohio, 43210, USA; 2 Department of Pharmaceutics, College of Pharmacy, The Ohio State University, Columbus, Ohio, 43210, USA; 3 Department of Veterinary Biosciences, The Ohio State University, Columbus, Ohio, 43210, USA; 4 Center for Retrovirus Research, The Ohio State University, Columbus, Ohio, 43210, USA; 5 Comprehensive Cancer Center, Arthur G. James Cancer Hospital and Solove Research Institute, The Ohio State University, Columbus, Ohio, 43210, USA

## 

Human T cell leukemia virus type 1 (HTLV-1), is complex deltaretrovirus linked to adult T-cell leukemia/lymphoma (ATL) and a variety of immune-mediated disorders. HTLV-1 encodes a nuclear localizing protein, p30, which selectively alters viral and cellular gene expression, activates G2-M cell cycle checkpoints, and is essential for viral spread. p30 interacts with key cellular proteins such as CBP/p300 and Myc/TIP60 to differentially modulate host and viral gene expression. We hypothesize that interaction of p30 with host cellular proteins modulates the cellular microenvironment to favor of viral spread. Herein we used immunoprecipitation, affinity pull-down of ectopically expressed p30 coupled with mass spectrometry to identify cellular binding partners of p30. Our data indicate that p30 specifically binds to cellular ataxia-telangiectasia mutated (ATM) and REGγ (a nuclear 20S proteasome activator). In conditions of genotoxic stress p30 expression was associated with reduced levels of ATM and increased cell survival. Knockdown or over expression of REGγ paralleled p30 expression suggesting an unexpected enhancement of p30 expression in the presence of REGγ. Finally, size exclusion chromatography revealed the presence of p30 in a high molecular weight complex along with ATM and REGγ. Current studies are focused on mapping regions critical for p30- REGγ binding and how this interaction may influence HTLV-1 transcription. Based on our findings we propose that HTLV-1 p30 interacts with ATM and REGγ to increase viral spread by facilitating cell survival.

